# Supplementary material for: B1 SINE-binding ZFP266 impedes mouse iPSC generation through suppression of chromatin opening mediated by reprogramming factors
Source: Nat Commun. 2023 Jan 30;14:488. doi: 10.1038/s41467-023-36097-9 (PMC9887000; doi:10.1038/s41467-023-36097-9)
Supplement: Supplementary file 1 — Supplementary Information [file 41467_2023_36097_MOESM1_ESM.docx]

**
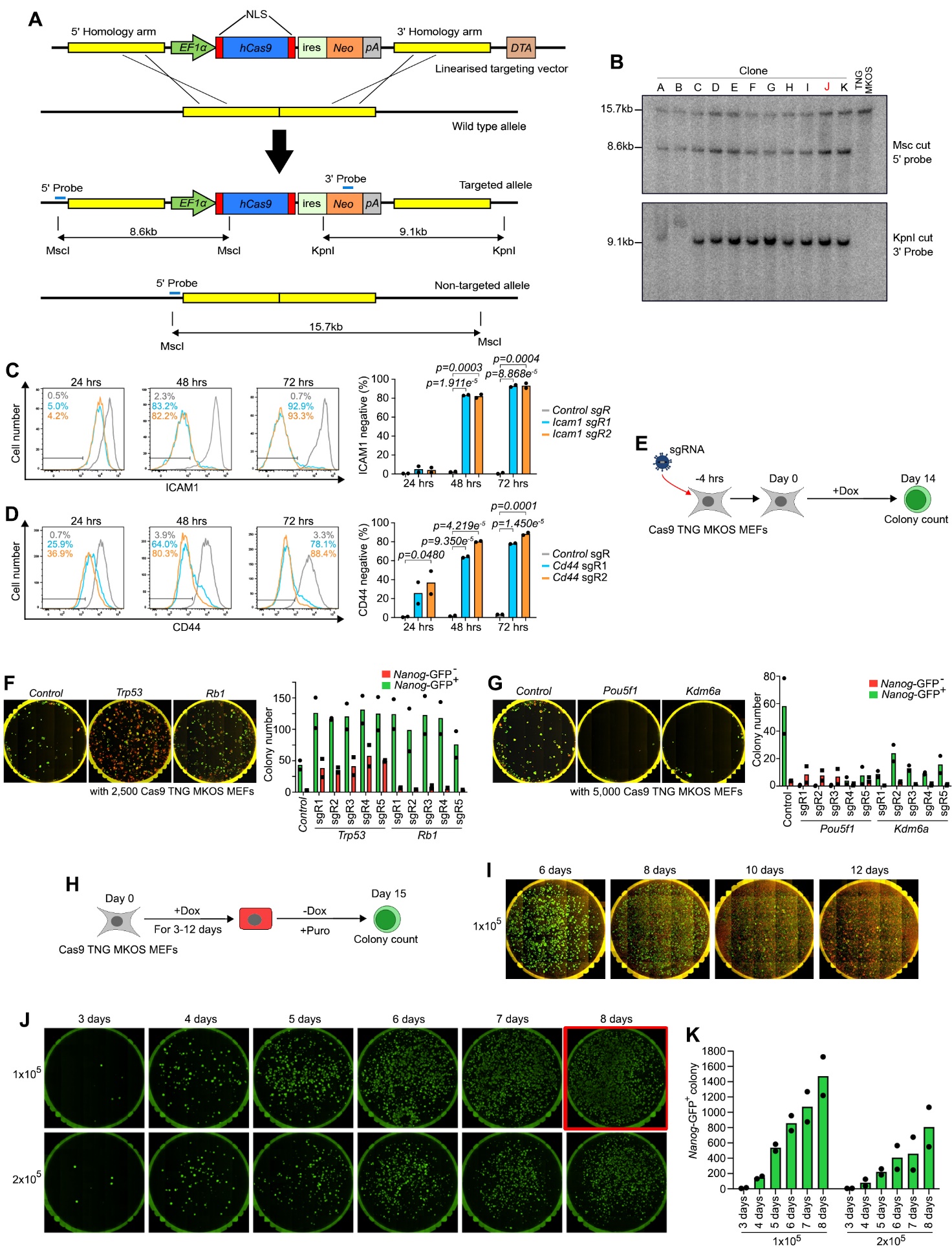
Supplementary Figure 1. Cas9 TNG MKOS MEFs as a tool for studying reprogramming. A.** Schematic diagram of the strategy to generate the Cas9 TNG MKOS ESC line. An *EF1α* promoter-driven *Cas9* expression cassette was inserted in the *Rosa26* locus of the TNG MKOS ESC line. NLS; Nuclear localisation signal, *hCas9*; Codon optimised SpCas9; ires; internal ribosome entry site, *neo*; Neomycin resistance gene, *pA;* polyadenylation signal, *DTA*; diphtheria toxin A. **B.** Southern blotting of selected G418 resistant clones. Clone J was used for all subsequent work. Southern blotting was carried out once. **C,D.** Loss of ICAM1 in TNG MKOS ESCs (**C**), and CD44 expression in Cas9 TNG MKOS MEFs (**D**), 24, 48, 72 hours after transduction of sgRNAs against *Icam1* and *Cd44*, respectively. Control sgRNA; non-targeting control sgRNA. The graph represents the average of 2 independent experiments. *p-*values are based on a one-tailed t-test. **E.** Schematic diagram of *Cas9* TNG MKOS MEF reprogramming with sgRNA expression. Cells were cultured in +dox for 14 days, starting 4 hours after sgRNA transduction. **F.** Cas9 TNG MKOS MEF reprogramming with *Trp53, Rb1* sgRNAs. The graph represents the mean of 2 independent experiments, with 2 technical replicates. **G.** Cas9 TNG MKOS MEF reprogramming with *Pou5f1, Kdm6a* sgRNA. The graph represents average of 2 independent experiments, with 2 technical replicates. **H.** Schematic diagram of the optimization strategy to obtain high numbers of *Nanog*-GFP+ iPSC colonies for the screen. Cas9 TNG MKOS MEFs seeded in the absence of feeders were cultured in +dox for 3-12 days and then in -dox +Puro until day 15 for colony counting**. I.** Day 15 whole-well images of reprogramming with +dox for 6, 8, 10, 12 days starting with 1x10^5^ Cas9 TNG MKOS MEFs on day 15. Keeping +dox conditions for over 8 days decreased *Nanog*-GFP+ iPSC colony number. Red; mOrange, Green; *Nanog*-GFP. **J.** Whole-well images of reprogramming with +dox for 3 to 8 days starting with 1x10^5^, 2x10^5^ Cas9 TNG MKOS MEFs. 8 days +dox starting with 1x10^5^ *Cas9* TNG MKOS MEFs (red square) was used for the screen. **K.** Quantification of Nanog-GFP+ colonies in J. The data represents average of 2 independent experiments, with 2 technical replicates. Source data are provided as a Source Data file.

**
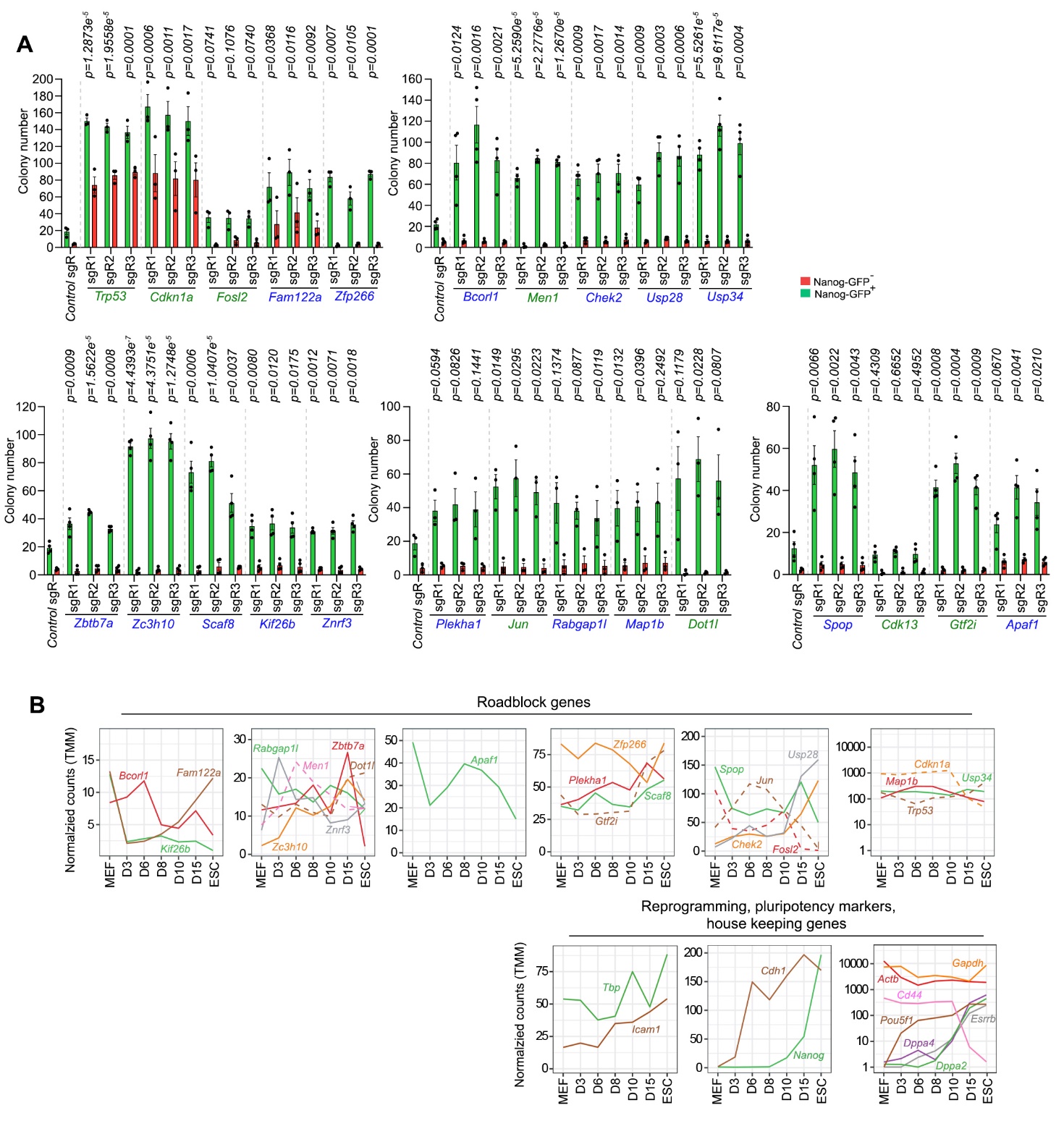
Supplementary Figure 2.** **Candidate gene KO reprogramming validation and expression pattern of those genes during reprogramming. A.** Number of *Nanog*-GFP^+^ and *Nanog*-GFP^-^ colonies in Cas9 TNG MKOS MEF reprogramming with sgRNA expression against the 24 roadblock genes. The data represents average of 3 (Trp53, Cdkn1a, Fosl2, Fam122a, Zfp266, Plekha1, Jun, Rabgap1l, Map1b, Dot1l) or 4 (Bcorl1, Men1, Chek2, Usp28, Usp34, Zbtb7a, Zc3h10, Sacf8, Kif26b, Znf3, Spop, Cdk13, Gtf2i, Apaf1) independent experiments, with 2 technical replicates. Error bars indicate SEM. *p-*values are based on an unpaired two tailed t test. **B.** Expression of the previously reported (dotted line) and other (unbroken line) roadblock genes (top panels), and reprogramming markers (*Icam1, Cdh1, Cd44*), pluripotency markers (*Nanog, Pou5f1, Esrrb, Dppa2, Dppa4*), and housekeeping genes (*Tbp, Gapdh, Actb*) (bottom panels) during reprogramming from GEO: GSE85178. The genes were grouped based on their highest expression levels. Source data are provided as a Source Data file.

**
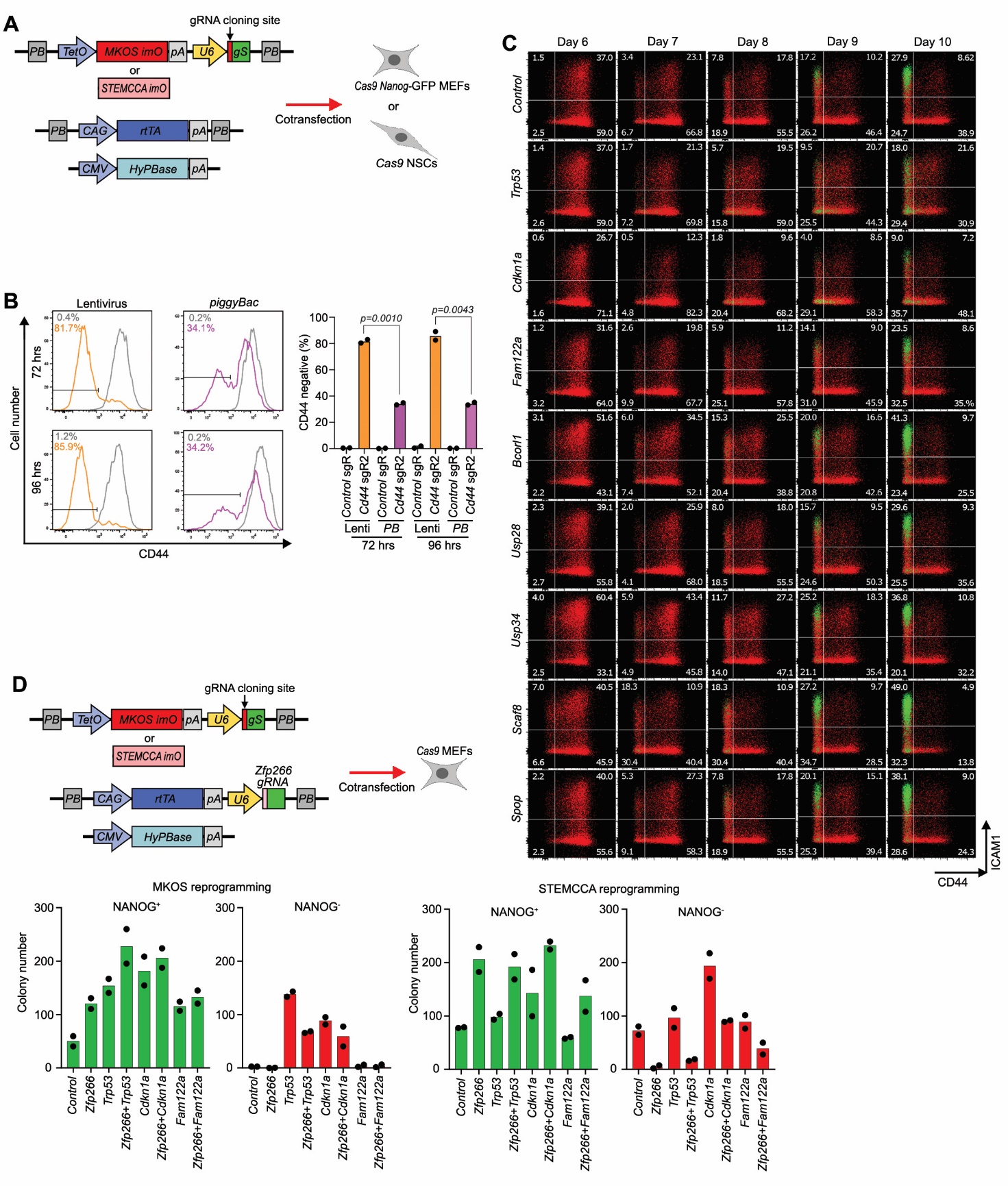
Supplementary Figure 3.** ***piggyBac* reprogramming with sgRNA expression and unaffected reprogramming kinetics by KO of roadblock genes. A.** Schematic diagram of *piggyBac* reprogramming with sgRNA expression. sgRNA sequences were cloned downstream of the U6 promoter in a *piggyBac* transposon carrying the dox-inducible *MKOS-ires-mOrange* (imO) or *STEMCCA-imO* reprogramming cassette. These *piggyBac* vectors were co-transfected with a *piggyBac* vector carrying a CAG promoter-driven rtTA expression cassette and a HyPBase expression vector in Cas9 expressing *Nanog*-GFP MEFs or NSCs. gS; scaffold sequence for sgRNA. **B.** Loss of CD44 by lentiviral (left) or *piggyBac* (right) delivery of a *Cd44* sgRNA in Cas9 *Nanog*-GFP MEFs. Only cells with the sgRNA expression vector were analysed based on fluorescent reporters on the vectors. The graph represents average of 2 independent experiments. *p*-values are based on a one-tailed t-test. **C**. CD44/ICAM/*Nanog*-GFP expression changes during reprogramming with sgRNA expression against roadblock genes. Data represent one of two experiments. Red; *Nanog*-GFP^-^ cells, Green; *Nanog*-GFP^+^ cells. **D.** *piggyBac*-mediated reprogramming with double KO of roadblock genes. sgRNAs against *Zfp266* and other roadblock genes were cloned into rtTA and reprogramming factor expression vectors, respectively. Transposons with indicated sgRNA(s) were transfected into Cas9 expressing MEFs. 14 days later numbers of NANOG^+^ and NANOG^-^ colonies were counted. Data represent an average of two independent experiments with 2 technical replicates. Source data are provided as a Source Data file.


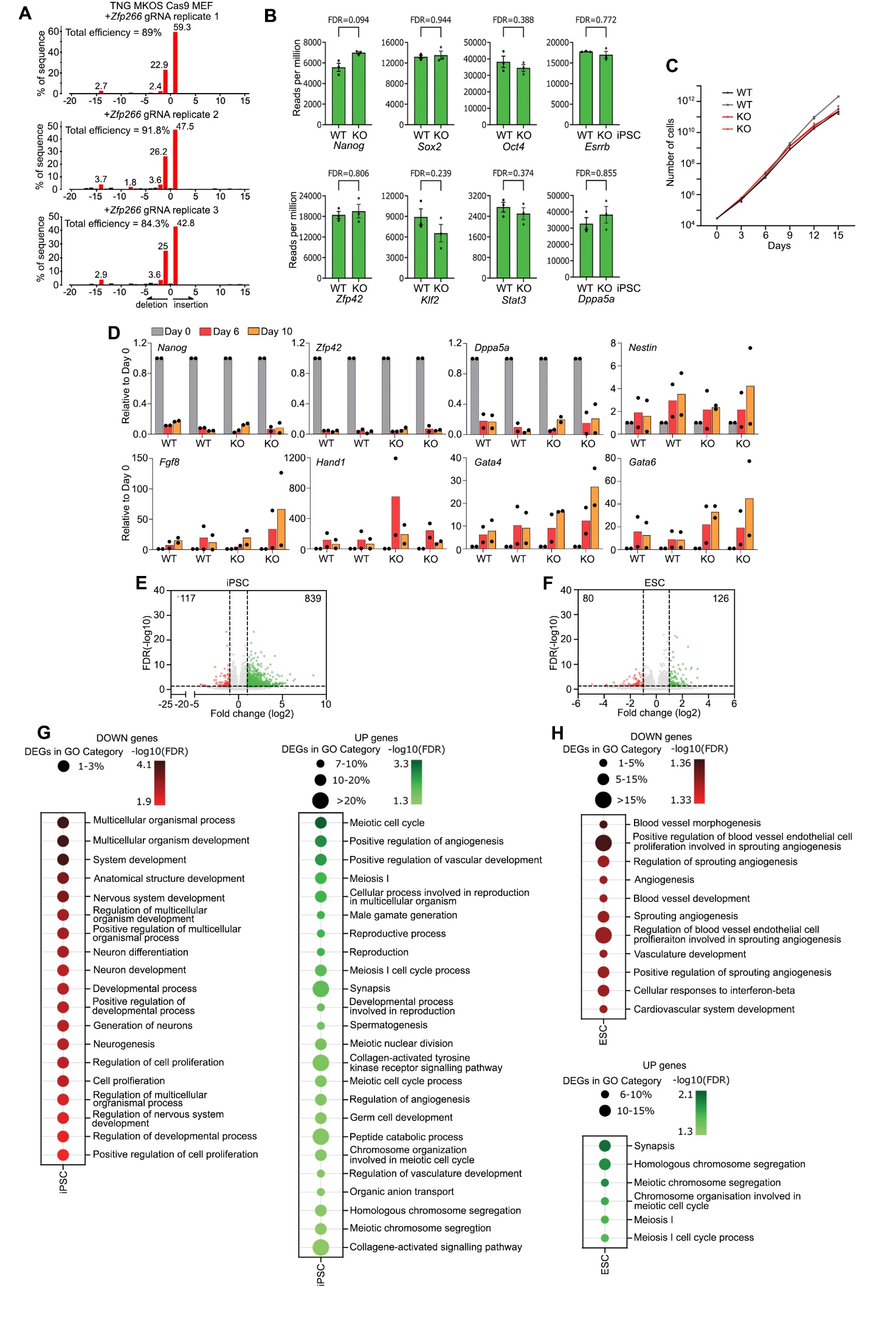


**Supplementary Figure 4. Characterization of ZFP266 KO MEFs, iPSCs and ESCs. A.** Indel mutation frequency estimated by a TIDE assay with *Zfp266* cDNA from Cas9 TNG MKOS MEFs with *Zfp266* sgRNA expression used for RNA-seq (*Zfp266* KO MEF samples). **B.** Pluripotency gene expression extracted from RNA-seq data of 3 independent iPSC clones established from *Zfp266* KO vs Wt reprogramming. Error bars indicate SEM. **C.** Proliferation of Wt and *Zfp266* KO iPSCs over 15 days. Data represent an average of 3 independent experiments. **D.** Expression of pluripotency (*Nanog, Zfp42, Dppa5a*), ectoderm (*Nestin*), mesoderm (*Fgf8*, *Hand1*), and endoderm (*Gata4, Gata6*) markers upon embryoid body differentiation of Wt and *Zfp266* KO iPSCs measured by RT-qPCR. The levels at day 6 and day 10 are represented relative to the levels at day 0. **E** and **F.** RNA-Seq volcano plot of iPSC clones established from *Zfp266* KO vs Wt reprogramming (E) and *Zfp266* KO vs Wt ESCs (F). Up-regulated and down-regulated genes in KO cells are shown to the right and left of the plot, respectively (cut-off FDR<0.05, log2FC>|1|). **G** and **H**. GO enrichment analysis of differentially expressed genes in *Zfp266* KO vs Wt iPSCs (G) and ESCs (H). Source data are provided as a Source Data file.

**
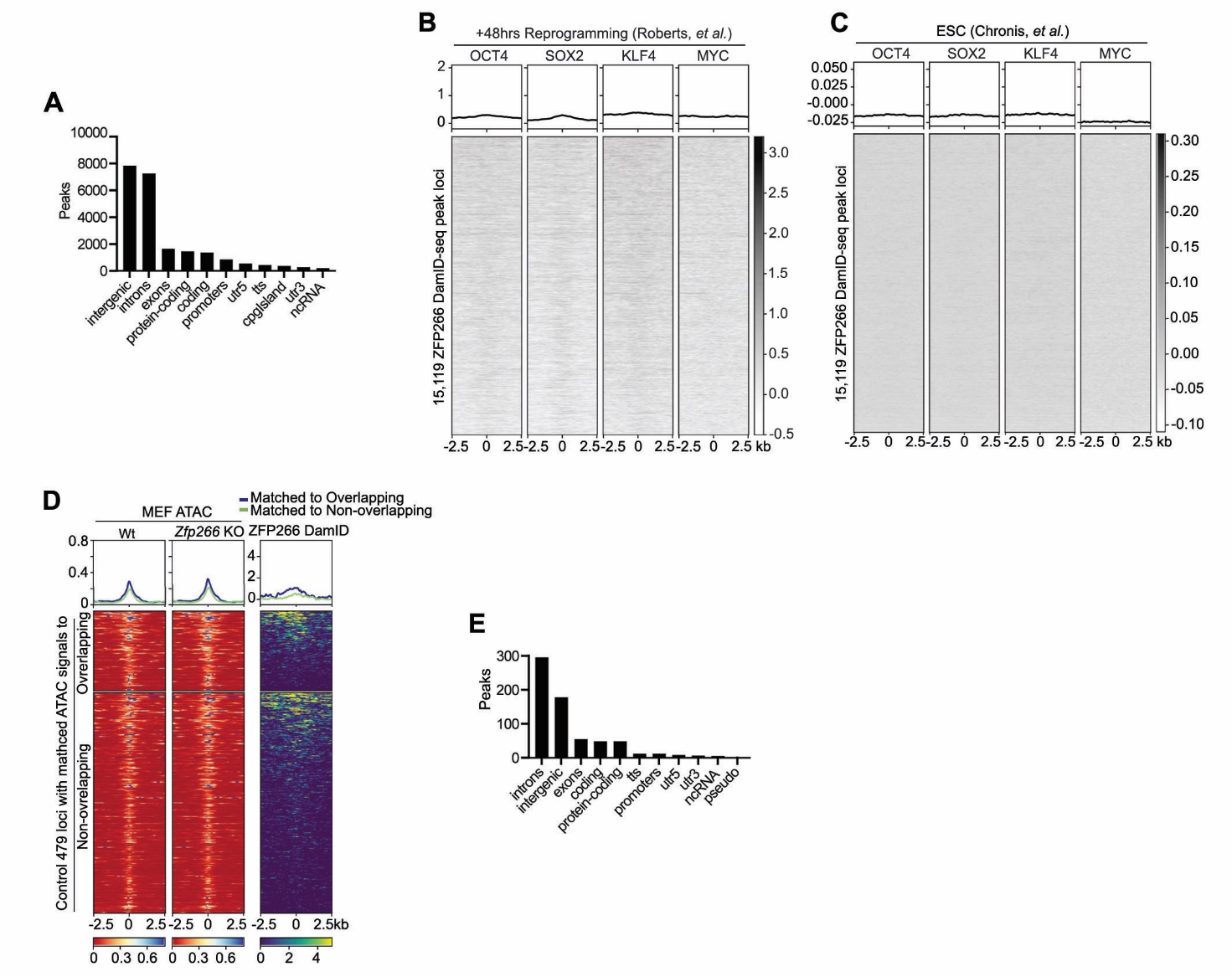
Supplementary Figure 5. No enrichment of OSKM binding at MEF ZFP266 DamID-seq peaks and ZFP266 DamID-seq signals at *Zfp266* KO MEF MOR control regions. A.** Number of ZFP266 DamID-seq peaks that overlap with regions with the indicated genomic annotations **B, C.** OSKM ChIP-Seq signals at 48 hours of reprogramming (**B**) and ESCs (**C**) at MEF ZFP266 DamID-seq peaks. Input subtracted ChIP-seq data are shown. **D**. ZFP266 DamID-seq signals at *Zfp266* KO MEF MOR control regions that have similar ATAC-signals to MORs. No ZFP266 DamID-seq signal enrichment is observed in these control loci. **E**. Number of *Zfp266* KO MEF MORs that overlap with regions with the indicated genomic annotations.**
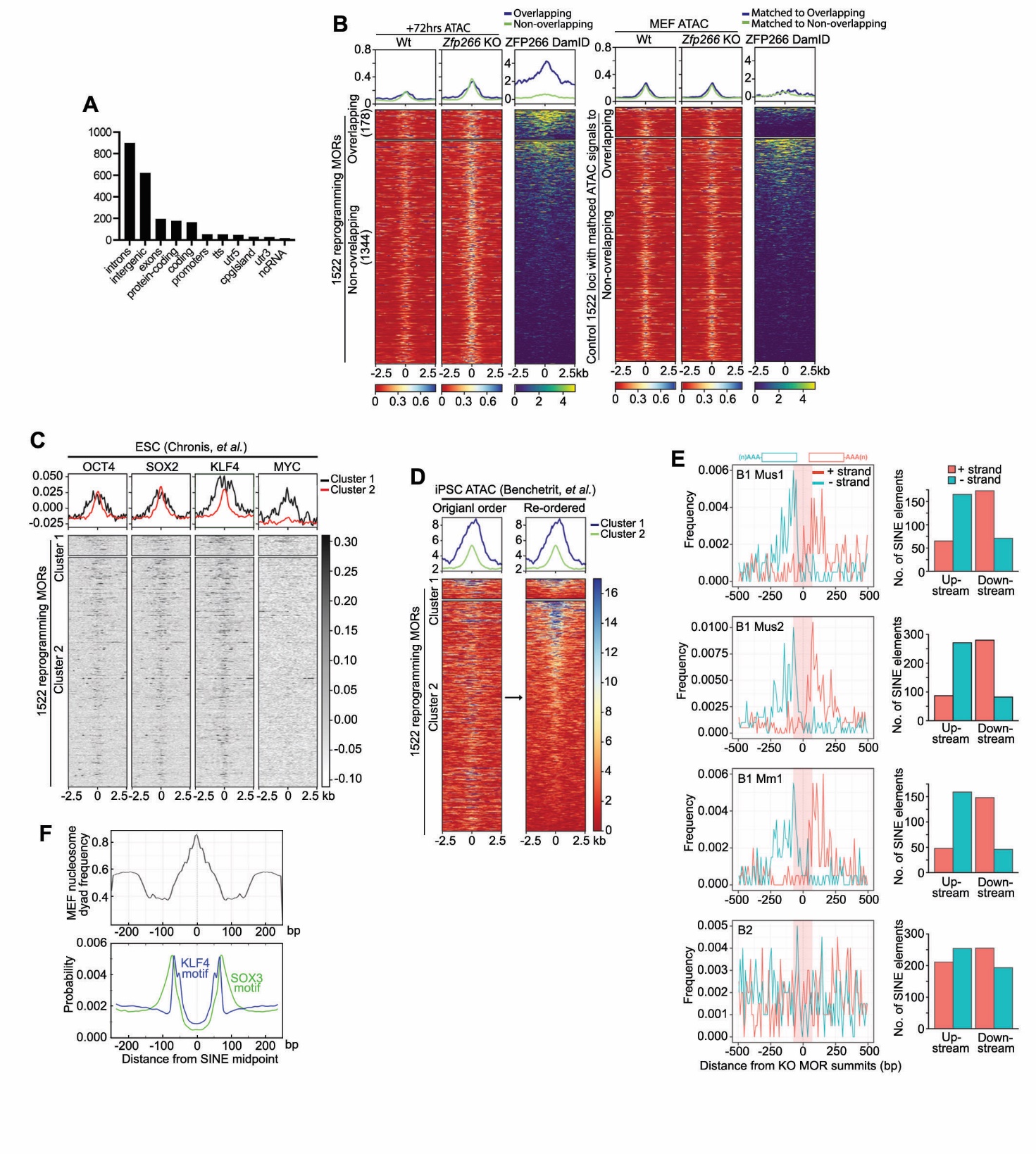
Supplementary Figure 6. No enrichment of MEF ZFP266 DamID-seq signals, but notable OSK binding in ESCs, at majority of *Zfp266* KO reprogramming MORs. A**. Number of *Zfp266* KO reprogramming MORs that overlap with regions with the indicated genomic annotations. **B**. MEF ZFP266 DamID-seq signals at *Zfp266* KO reprogramming MORs (left) and control loci with matched chromatin accessibility (right). The DamID-seq signals at non-overlapping MORs is equivalent to those at matched control loci, unlike overlapping MORs, confirming only ~10% of reprogramming MORs are bound by ZFP266 in MEFs. **C.** OSKM ChIP-Seq signals in ESCs at *Zfp266* KO reprogramming MOR loci. Input subtracted ChIP-seq data are shown. **D**. ATAC-seq signals in iPSCs at *Zfp266* KO reprogramming MOR loci, in the descending order of *Zfp266* KO reprogramming ATAC-seq signals (left) or iPSC ATAC-seq signals (right). **E**. Distribution of B1 SINE subfamilies and B2 SINEs within *Zfp266* KO reprogramming MORs. Graphs on right show the actual numbers of SINEs located on the plus or minus strand either upstream or downstream of the MOR summits. **F**. Nucleosome dyad frequency at all B1 SINEs using MNase-seq data with MEFs (GSM1004654) (top), and KLF4 and SOX3 motif enrichment at the same regions (bottom).

**
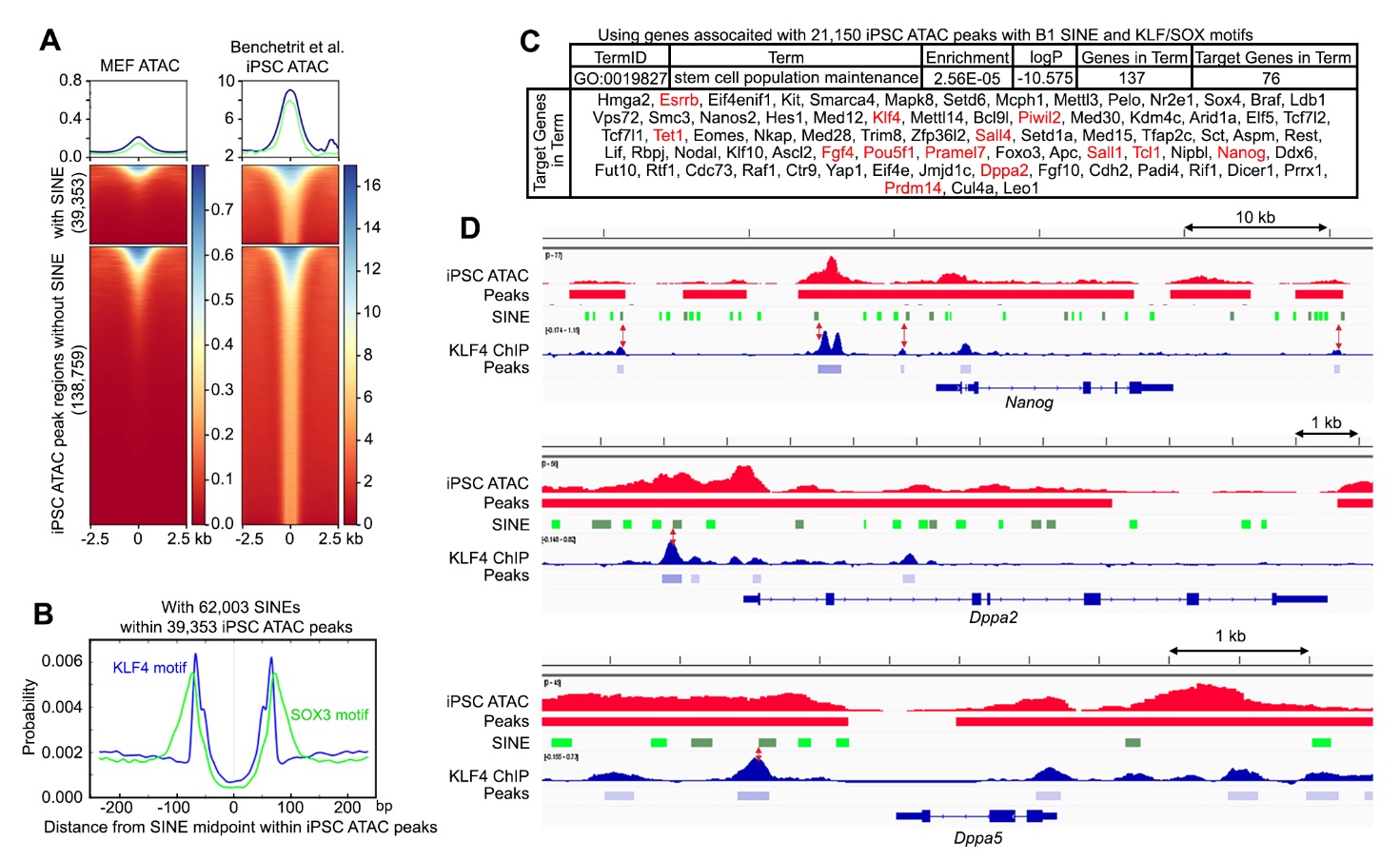
Supplementary Figure 7. B1 SINEs in iPSC open chromatin regions. A.** Of 178,112 iPSC ATAC-peaks, 22% (39,353) has at least one B1 SINE, majority of which are closed in MEFs. **B.** Those B1 SINEs at iPSC ATAC-peaks have KLF4 and SOX3 motif enrichment at the head and the tail of SINE. **C.** Gene associated with 12,150 iPSC ATAC-seq peaks with at least one B1 SINE and KLF/SOX motif are enriched in genes with a GO term “stem cell population maintenance”. Well known pluripotency associated genes are highlighted in red. **D.** Pluripotency gene loci with iPSC ATAC-seq peaks (red), B1 SINE (green) and KLF4 ChIP-seq signals (Blue). B1 SINE with KLF4 motif is indicated in dark green, and proximity of B1 SINEs to KLF4 ChIP-seq peaks is indicated in red arrows. Input subtracted ChIP-seq data are shown.


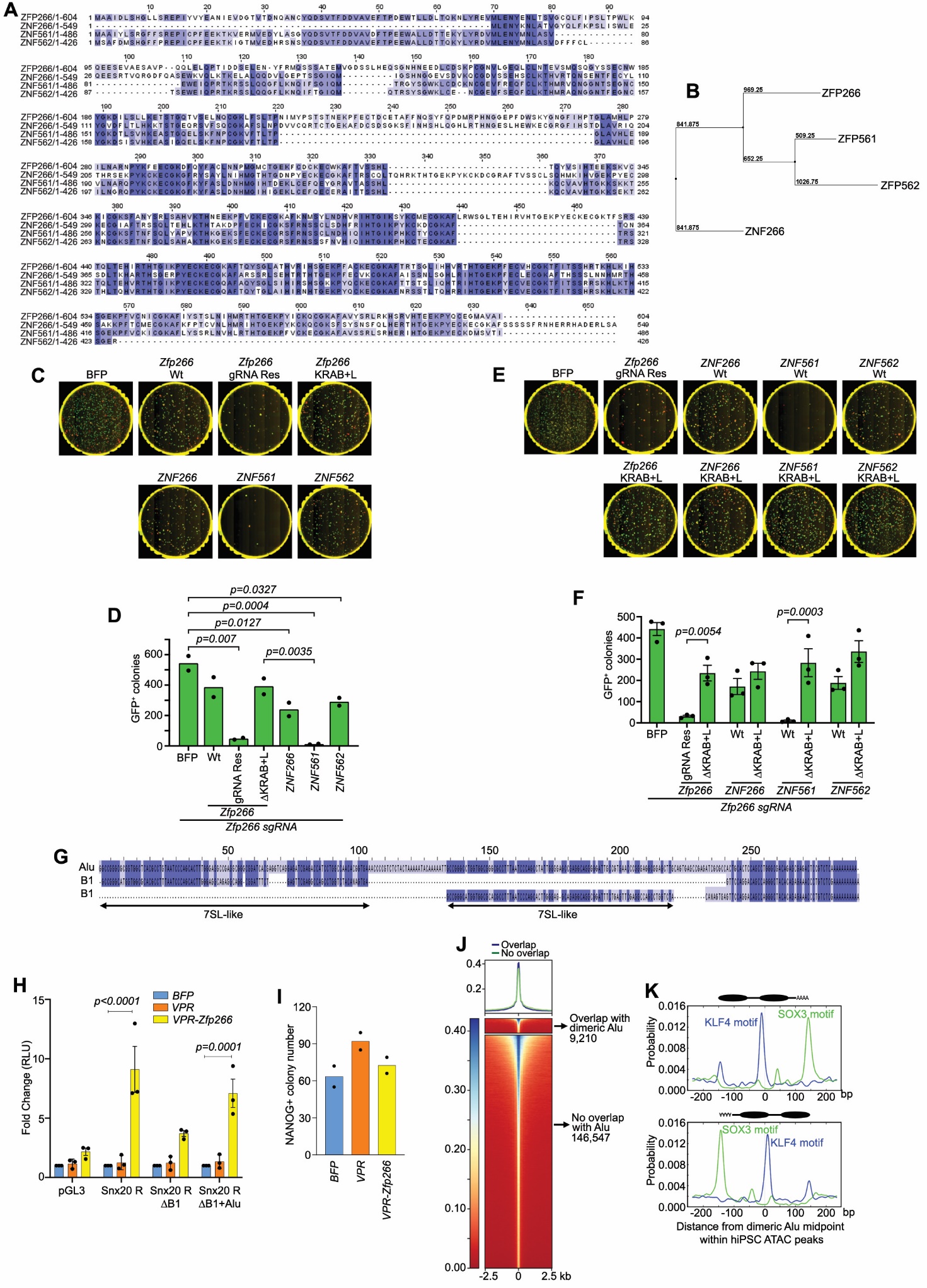


**Supplementary Figure 8. Human orthologue of mouse ZFP266. A.** Amino acid sequence alignment of mouse ZFP266, human ZNF266, ZNF561 and ZNF562 using Jalview **B.** Neighbour joining Tree of mouse ZFP266, human ZNF266, ZNF561 and ZNF562. **C** and **D.** Cas9 *Nanog*-GFP MEF reprogramming with MKOS piggyBac transposons, *Zfp266* sgRNA expression as well as cDNA overexpression of *BFP*, wild-type *Zfp266* (Wt), sgRNA resistant *Zfp266* (gRNA Res), sgRNA resistant *Zfp266* with KRAB domain deletion (ΔKRAB+L), human *ZNF266*, *ZNF561* or *ZFN562*, imaged at day 15. Red; mOrange, Green; *Nanog*-GFP (C). Mean *Nanog*-GFP+ colony numbers of C (D). *p*-values are based on a one-way ANOVA test. **E** and **F.** Cas9 *Nanog*-GFP MEF reprogramming with MKOS piggyBac transposons, *Zfp266* sgRNA expression as well as cDNA overexpression of *BFP*, sgRNA resistant *Zfp266* (gRNA Res), sgRNA resistant *Zfp266* with KRAB domain deletion (ΔKRAB+L), human *ZNF266*, *ZNF561* or *ZFN562* with (ΔKRAB+L) or without (Wt) KRAB domain deletion, imaged at day 15. Red; mOrange, Green; Nanog-GFP (E). Mean *Nanog*-GFP+ colony numbers of E (F). Error bars indicate SEM, *p*-values are based on a one-way ANOVA test. **G.** DNA sequence alignment of mouse B1 SINE and human Alu. **H.** Luciferase reporter assay with an empty reporter vector (pGL3) or vectors containing *Snx20* regulatory region (Snx20 R), *Snx20* regulatory region with B1 SINE deletion (Snx20 R ΔB1) and *Snx20* regulatory region where B1 SINE as substituted with an Alu sequence (Snx20 R ΔB1+Alu), co-transfected with either *BFP*, *VPR* only or *VPR-Zfp266* expression vectors in HEK293 cells. Data are mean values of three independent experiments and error bars represent SEM. *p*-values are obtained using a two-way ANOVA test. **I.** Human dermal fibroblast reprogramming using lentiviral vectors expressing *OCT4, SOX2, KLF4* and *MYC*, with *VPR-ZFP266*. NANOG+ colony numbers were counted on day 21 of reprogramming. Graph represents averages of 2 technical replicates. **J.** Heatmap of human ESC ATAC-seq peak regions with (top) or without (bottom) overlap with Alu. **K.** KLF4 and SOX3 motif enrichment at Alu within human ESC ATAC-seq peaks. Alu on the top and bottom strand are analysed separately. Source data are provided as a Source Data file.
