# Supplementary material for: B1 SINE-binding ZFP266 impedes mouse iPSC generation through suppression of chromatin opening mediated by reprogramming factors
Source: Nat Commun. 2023 Jan 30;14:488. doi: 10.1038/s41467-023-36097-9 (PMC9887000; doi:10.1038/s41467-023-36097-9)
Supplement: Supplementary file 3 — Description of Additional Supplementary Files [file 41467_2023_36097_MOESM3_ESM.pdf]

Description of Additional Supplementary Files:

Supplementary Data 1: sgRNA read counts from the genome-wide KO screen with 3 technical replicates

Supplementary Data 2: Statistical analysis results of the KO screen with MAGeCK

Supplementary Data 3: Normalized read counts of RNA-seq during reprogramming with and without Zfp266 KO

Supplementary Data 4: Differentially expressed genes (DEG) between wild-type and Zfp266 KO MEFs. The differential expression analysis was performed using DESeq2 which uses a Wald test (a two-sided test) to detect statistically significant differentially expressed genes. It also uses the Benjamini-Hochberg method to adjust for multiple testing.

Supplementary Data 5: Differentially expressed genes (DEG) between wild-type and Zfp266 KO MEF reprogramming day 3. The differential expression analysis was performed using DESeq2 which uses a Wald test (a two-sided test) to detect statistically significant differentially expressed genes. It also uses the Benjamini-Hochberg method to adjust for multiple testing.

Supplementary Data 6: Differentially expressed genes (DEG) between wild-type and Zfp266 KO MEF reprogramming day 5. The differential expression analysis was performed using DESeq2 which uses a Wald test (a two-sided test) to detect statistically significant differentially expressed genes. It also uses the Benjamini-Hochberg method to adjust for multiple testing.

Supplementary Data 7: Differentially expressed genes (DEG) between wild-type and Zfp266 KO MEF reprogramming day 7. The differential expression analysis was performed using DESeq2 which uses a Wald test (a two-sided test) to detect statistically significant differentially expressed genes. It also uses the Benjamini-Hochberg method to adjust for multiple testing.

Supplementary Data 8: Differentially expressed genes (DEG) between wild-type and Zfp266 KO ESCs. The differential expression analysis was performed using DESeq2 which uses a Wald test (a two-sided test) to detect statistically significant differentially expressed genes. It also uses the Benjamini-Hochberg method to adjust for multiple testing.

Supplementary Data 9: Peak annotation of ZFP266 DamID in MEFs

Supplementary Data 10: Differentially opened regions from ATAC KO vs wild-type MEF ATAC-seq. The differential open region analysis was performed using DiffBind which internally uses DESeq2. Thus, statistically significant differentially open regions was detected by a Wald test (a two-sided test) and the Benjamini-Hochberg method for multiple testing.

Supplementary Data 11: Differentially opened regions from ATAC KO vs wild-type reprogramming day 3 ATAC-seq. The differential open region analysis was performed using DiffBind which internally uses DESeq2. Thus, statistically significant differentially open regions was detected by a Wald test (a two-sided test) and the Benjamini-Hochberg method for multiple testing.

Supplementary Data 12: Reprogramming MOR annotation

Supplementary Data 13: Plasmid information
