# Supplementary material for: B1 SINE-binding ZFP266 impedes mouse iPSC generation through suppression of chromatin opening mediated by reprogramming factors
Source: Nat Commun. 2023 Jan 30;14:488. doi: 10.1038/s41467-023-36097-9 (PMC9887000; doi:10.1038/s41467-023-36097-9)
Supplement: Supplementary file 17 — Reporting summary [file 41467_2023_36097_MOESM17_ESM.pdf]

nature portfolio

Corresponding author(s): Keisuke Kaji

Last updated by author(s): Dec 13, 2022

Reporting Summary

Nature Portfolio wishes to improve the reproducibility of the work that we publish. This form provides structure for consistency and transparency in reporting. For further information on Nature Portfolio policies, see our Editorial Policies and the Editorial Policy Checklist.

Statistics

For all statistical analyses, confirm that the following items are present in the figure legend, table legend, main text, or Methods section.

n/a	Confirmed	
☐	☒	The exact sample size (*n*) for each experimental group/condition, given as a discrete number and unit of measurement
☐	☒	A statement on whether measurements were taken from distinct samples or whether the same sample was measured repeatedly
☐	☒	The statistical test(s) used AND whether they are one- or two-sided<br>*Only common tests should be described solely by name; describe more complex techniques in the Methods section.*
☒	☐	A description of all covariates tested
☒	☐	A description of any assumptions or corrections, such as tests of normality and adjustment for multiple comparisons
☐	☒	A full description of the statistical parameters including central tendency (e.g. means) or other basic estimates (e.g. regression coefficient) AND variation (e.g. standard deviation) or associated estimates of uncertainty (e.g. confidence intervals)
☐	☒	For null hypothesis testing, the test statistic (e.g. *F*, *t*, *r*) with confidence intervals, effect sizes, degrees of freedom and *P* value noted<br>*Give P values as exact values whenever suitable.*
☒	☐	For Bayesian analysis, information on the choice of priors and Markov chain Monte Carlo settings
☒	☐	For hierarchical and complex designs, identification of the appropriate level for tests and full reporting of outcomes
☒	☐	Estimates of effect sizes (e.g. Cohen's *d*, Pearson's *r*), indicating how they were calculated

Our web collection on statistics for biologists contains articles on many of the points above.

Software and code

Policy information about availability of computer code

Data collection	Flowjo v10, Celigo (5.2.0.0)
Data analysis	SAMtools (1.3), BEDtools (2.29.2), DESeq2 (3.2), MACS2 (2.1.0.20151222), Daim (https://github.com/ndomon/DAIM), HOMER (v4.11), deepTools (v3.3.2) , MEME suite (5.1.1), Jalview (2.11.2.5), MAGeCK (0.4.2)

For manuscripts utilizing custom algorithms or software that are central to the research but not yet described in published literature, software must be made available to editors and reviewers. We strongly encourage code deposition in a community repository (e.g. GitHub). See the Nature Portfolio guidelines for submitting code & software for further information.

Data

Policy information about availability of data

All manuscripts must include a data availability statement. This statement should provide the following information, where applicable:

- Accession codes, unique identifiers, or web links for publicly available datasets
- A description of any restrictions on data availability
- For clinical datasets or third party data, please ensure that the statement adheres to our policy

Raw and processed RNA-seq, DamID-seq, ATAC-seq data are available at GSE198166 from 19th December 2022.
ATAC-seq data of iPSCs were retrieved from GSE98124.
ChIP-seq data of ESCs and MEFs in early reprogramming at 48 hr were retrieved from GSE90895 and GSE168142.

Micrococcal nuclease-sequencing (MNase-seq) data of MEF was retrieved from GSE40896.

Human research participants

Policy information about studies involving human research participants and Sex and Gender in Research.

Reporting on sex and gender	N/A
Population characteristics	N/A
Recruitment	N/A
Ethics oversight	N/A

Note that full information on the approval of the study protocol must also be provided in the manuscript.

Field-specific reporting

Please select the one below that is the best fit for your research. If you are not sure, read the appropriate sections before making your selection.

☒ Life sciences ☐ Behavioural & social sciences ☐ Ecological, evolutionary & environmental sciences

For a reference copy of the document with all sections, see nature.com/documents/nr-reporting-summary-flat.pdf

Life sciences study design

All studies must disclose on these points even when the disclosure is negative.

Sample size	Experiments were performed with biological replicates when available as stated in the manuscript. Sample size calculation was not carried out preceding the experiments, but sample sizes were determined based on previous experiences. In general, 2-3 technical replicates allowed us to make sure the experiments have minimal technical variations, and we focused on the data which made statistical differences within 2-3 independent experiments. We aimed to set up different experiments to variate the data when possible instead of repeating the same experiments.
Data exclusions	No data exclusion.
Replication	At least 2 technical replicates were included in all experiments. As a nature of the experiments, some of the variations were large. Statistical tests showed which data were reproducible.
Randomization	For sequencing experiments, samples were bar-coded randomly and sequenced all together in the same lane in oder to avoid batch effects. For iPSC colony counting, we used an image analysis tool in Celigo, thus randomization of samples were not required.
Blinding	Counting iPSC colony numbers were carried out using a whole well imaging machine, Celigo, in order not to have a bias of the examiner. Luciferase assays and RT-PCR does not require blinding as the measurements are given by the machines.

Reporting for specific materials, systems and methods

We require information from authors about some types of materials, experimental systems and methods used in many studies. Here, indicate whether each material, system or method listed is relevant to your study. If you are not sure if a list item applies to your research, read the appropriate section before selecting a response.

Materials & experimental systems

n/a	Involved in the study	
☐	☒	Antibodies
☐	☒	Eukaryotic cell lines
☒	☐	Palaeontology and archaeology
☐	☒	Animals and other organisms
☒	☐	Clinical data
☒	☐	Dual use research of concern

Methods

n/a	Involved in the study	
☒	☐	ChIP-seq
☐	☒	Flow cytometry
☒	☐	MRI-based neuroimaging

Antibodies

Antibodies used	ICAM1-biotin (13-0541-82, eBioscience, 1/100)<br>CD44-APC (17-0441-82, eBioscience, 1/300)<br>streptavidin-PE-Cy7 (25-4317-82, eBioscience, 1/1500)<br>NANOG (eBioMLC-51, Thermofisher Scientific, 1/1000)<br>AlexaFluor488 conjugated secondary antibody (A-21208, Invitrogen, 1/1000)
Validation	Using those antibodies, we confirmed that; ES cells are 100% positive for ICAM1, 100% negative for CD44, majority of ES cells are NANOG positive, MEFs are 100% positive for CD44, 100% negative for NANOG, and ~50% of MEFs are negative for ICAM1.

Eukaryotic cell lines

Policy information about cell lines and Sex and Gender in Research

Cell line source(s)	Cas9 TNG MKOS ES line was generated from mouse ES cell line, E14tg2a, with 129 mouse genetic background. HEK293 cells (human embryonic kidney epithelial cells) are purchased from Merck.
Authentication	Pluripotency of Cas9 TNG MKOS ES lines has been confirmed by generating chimeric mice.
Mycoplasma contamination	Mycoplasma contamination-free state has been confirmed by a qPCR mycoplasma test.
Commonly misidentified lines<br>(See ICLAC register)	No commonly misidentified cell lines were used in this study.

Animals and other research organisms

Policy information about studies involving animals; ARRIVE guidelines recommended for reporting animal research, and Sex and Gender in Research

Laboratory animals	Mouse
Wild animals	Not used.
Reporting on sex	8-10 week, B6CBAF1 female mice were used to collect embryos for morula aggregation. generate chimeric embryos and isolate fibroblasts.
Field-collected samples	No field collected samples were used in the study
Ethics oversight	Experiments with animals have been approved by the University of Edinburgh Animal Welfare and Ethical Review Body and carried out under licenses authorized by the Home Office in UK.

Note that full information on the approval of the study protocol must also be provided in the manuscript.

Flow Cytometry

Plots

Confirm that:

☒ The axis labels state the marker and fluorochrome used (e.g. CD4-FITC).

☒ The axis scales are clearly visible. Include numbers along axes only for bottom left plot of group (a 'group' is an analysis of identical markers).

☒ All plots are contour plots with outliers or pseudocolor plots.

☒ A numerical value for number of cells or percentage (with statistics) is provided.

Methodology

Sample preparation	Cells harvested at different time points of reprogramming were stained in FACS buffer for 30 min at 4°C and washed with FACS buffer prior to acquisition with LSR Fortessa (BD Biosciences) cytometer. The following antibodies from eBioscience were used: ICAM1-biotin (13-0541-82; Dilution: 1/100), CD44-APC (17-0441-82; Dilution 1/300), streptavidin-PE-Cy7 (25-4317-82; Dilution: 1/1500). Dead cells were excluded using LIVE/DEAD™ Fixable Near-IR Dead Cell Stain Kit (ThermoFisher Scientific, Dilution: 1/1500).
Instrument	LSR Fortessa, AriaII (BD biosciences)
Software	Flowjo v10

Cell population abundance	For RNA-seq, mOrange+ reprogramming factor expressing cells were flow-sorted. Those cells were typically 10-40%, and purity after sorting was >90%. For DamID-seq, plasmid transfected GFP+ cells were 10-50% of entire samples and purity after sorting was >90%.
Gating strategy	Using SSC/FSC/Draq7 staining, only live singlet cells were used for further analysis/sorting. Positve/negative boundaries were set based on unstained or fluorescence protein non-expressing cells.

☒ Tick this box to confirm that a figure exemplifying the gating strategy is provided in the Supplementary Information.
